# Potential of inclusion bodies for disease control in aquaculture and livestock

**DOI:** 10.1016/j.vas.2026.100820

**Published:** 2026-08-15

**Authors:** Daniela Valentina López, José Gallardo-Matus, Débora Torrealba

**Affiliations:** a Laboratorio de Genética y Genómica Aplicada, Escuela de Ciencias del Mar, Pontificia Universidad Católica de Valparaíso, Avenida Universidad 330, Valparaíso, 2373223, Chile; b Programa de Doctorado en Biotecnología Pontificia Universidad Católica de Valparaíso/Universidad Técnica Federico Santa María, Valparaíso, Chile; c Laboratorio de Nanobiotecnología e Inmunología Marina, Instituto de Ciencias de la Ingeniería, Universidad de O’Higgins, Avenida Libertador Bernardo O’Higgins 611, Rancagua, 2841959, Chile

**Keywords:** Recombinant protein, Therapeutic, Immune system, Pathogens, Animals

## Abstract

Inclusion bodies are insoluble nanostructured recombinant proteins that have emerged as innovative biomaterials for disease control in animal production. This systematic literature review aimed to explore the use of inclusion bodies for disease control in aquaculture and livestock. We conducted a literature search in four databases: PubMed, Web of Science, ScienceDirect and Scopus. Of the 3139 research articles retrieved, only 16 met our eligibility criteria: 6 for aquaculture, including studies in rainbow trout, senegalese sole, and zebrafish, and 10 for livestock, including studies in cattle, sheep, swine, and mouse models. The proteins produced in these studies were mainly host immune system proteins and pathogen antigenic proteins. The diseases or pathogens potentially controlled by inclusion bodies in aquaculture were *Pseudomonas aeruginosa, Mycobacterium marinum*, Spring viremia of carp virus and Viral nervous necrosis virus. Conversely, the diseases or pathogens evaluated to be controlled by inclusion bodies in livestock were mastitis, *Fasciola hepatica, Lawsonia intracellularis*, and *Vibrio sp*. The administration strategies varied greatly, including intra-mammary gland infusion, nasal suspension, gastric injection, esophageal intubation, intramuscular injection, and oral administration. Regardless of administration route, these biomaterials consistently activated immune responses through two mechanisms: modulation of the host immune system via immunostimulatory proteins, and activation of adaptive immunity via antigenic proteins. Although promising, widespread adoption remains limited by technological challenges including purification standardization, batch variability, and lack of studies in commercially relevant species. These findings establish a robust evidence base to inform the development of IB-based interventions in animal health and guide future research directions.

## Graphical abstract

**Figure:**
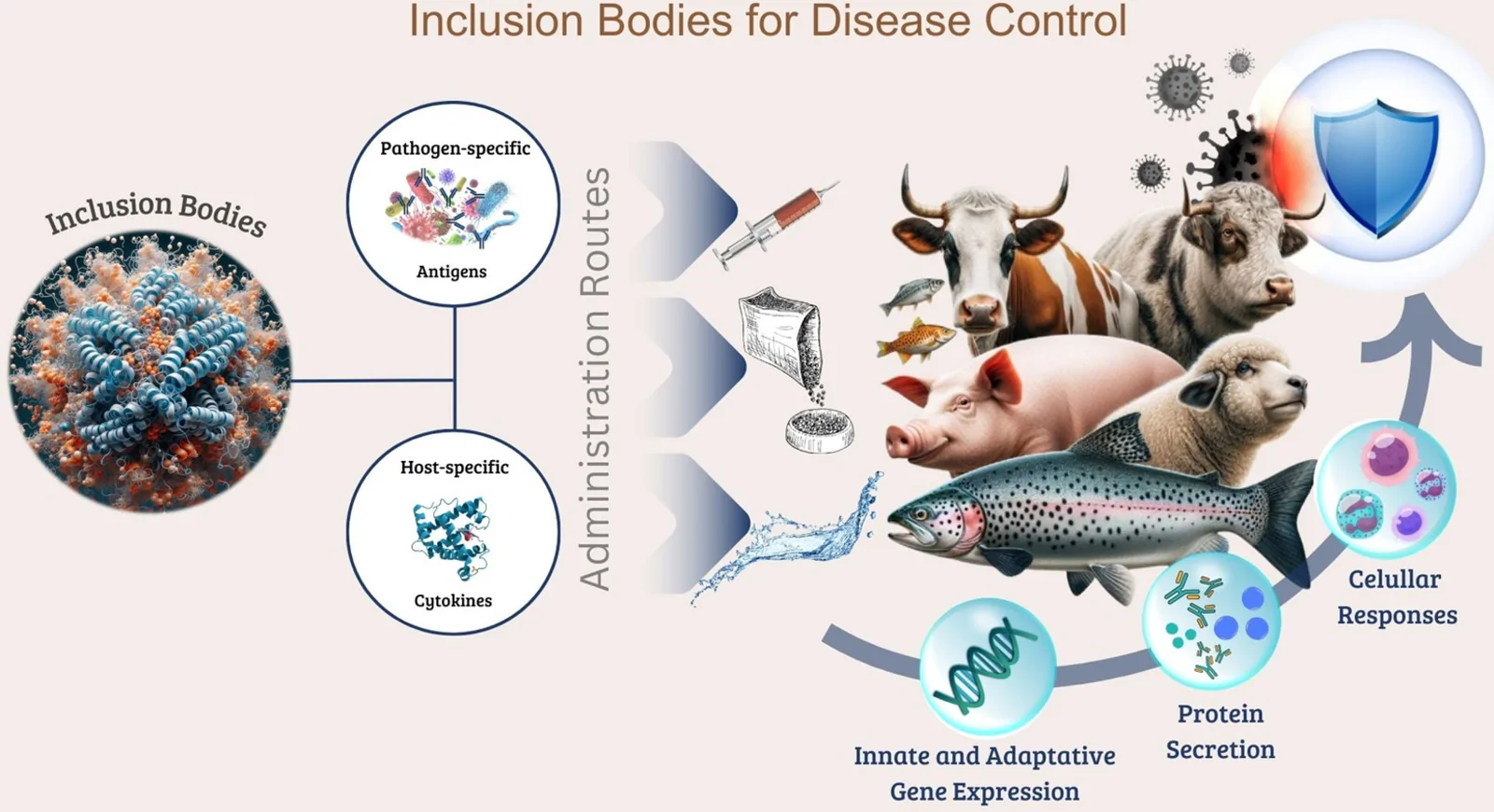

**List of Abbreviations**
Camp1: cathelicidin antimicrobial peptide
CCL4: Chemokine (C-C motif) ligand 4
CD4: Cluster of Differentiation 4
Cox2: cyclooxygenase-2
FESEM: Field Emission Scanning Electron Microscopy
GRAS: Generally Recognized as Safe
IBs: inclusion bodies
IFNγ: interferon gamma
IgG: immunoglobulin G
IgM: immunoglobulin M
IL1β: interleukin 1 beta
IL6: interleukin 6
IL8: interleukin 8
IL12: interleukin 12
LPS: Lipopolysaccharides
MMP9: Matrix Metalloproteinase-9
Omp: Outer Membrane Protein
PICOS: Population, Intervention, Comparator, Outcomes, Study design
PRISMA: Preferred Reporting Items for Systematic Reviews and Meta-analyses
RPS: Relative Percentage of Survival
SVCV: spring viremia of carp virus
TLR4: Toll-Like Receptor 4
Th1: T helper 1
TNFα: Tumor Necrosis Factor alpha
VHSV: viral hemorrhagic septicemia virus

## 1 Introduction

One of the main global challenges is achieving sufficient animal protein production to feed a growing population, estimated to reach 10 billion people by 2050 (Fróna et al., 2019). This challenge has positioned livestock as the primary source of animal-based proteins globally, with 316 million tonnes produced in 2022 (FAO, 2023), while simultaneously driving substantial growth in aquaculture, which reached 185.4 million tonnes of aquatic animals in the same year (FAO, 2024). However, meeting the increasing demand for animal-based food requires optimizing production processes, particularly through reducing the productive losses associated with diseases (Hennessy & Marsh, 2021; FAO, 2024). Furthermore, climate change is exacerbating this scenario, as rising temperatures and extreme weather events are increasing the incidence and geographical spread of infectious diseases in both aquaculture and livestock (Bett et al., 2017; Okon et al., 2024), further disease control strategies in animal production rely heavily on antibiotics (Gaballah et al., 2021; Ferri et al., 2022; Bondad-Reantaso et al., 2023; Wang et al., 2023; Jiang et al., 2024).

Livestock production commonly employs antimicrobials such as ampicillin, gentamicin, and erythromycin, while aquaculture frequently relies on oxytetracycline, florfenicol, and sulfadiazine for disease control (Bondad-Reantaso & Subasinghe, 2012; Lozano et al., 2018; Lulijwa et al., 2019). In most developed countries, the livestock sector alone consumes between 50%−80% of all antibiotics produced (Cully, 2014; Vidovic & Vidovic, 2020; Haulisah et al., 2021). Despite antimicrobials causing significant environmental problems through residue accumulation and the emergence of resistant bacteria (Higuera-Llantén et al., 2018; Guo et al., 2021), their complete elimination from animal production remains unlikely due to demonstrated benefits (Callaway et al., 2021). This scenario underscores the urgent need for integrated One Health approaches that address antimicrobial use across animal, human, and environmental health simultaneously (Cella et al., 2023).

To address these concerns, alternatives to antimicrobials have been developed, including prebiotics, probiotics, organic acids, phytobiotic products, exogenous enzymes, phage therapy, vaccines, immunostimulants, antimicrobial peptides, and bacteriocins (Lagua & Ampode, 2021; Enayet Kabir et al., 2021; Ampode & Asimpen, 2021). However, the development of these alternatives faces constraints due to lower profitability compared to human treatments, attributed to smaller markets and reduced pricing. This economic limitation restricts research and development investment, elevating product costs and limiting accessibility for cost-conscious primary producers (Meeusen et al., 2007; Covarrubias et al., 2012). Consequently, innovation in alternatives that are both efficient and cost-effective is essential for successful application in farmed animals.

Among various antimicrobial alternatives, biotechnological approaches have gained particular attention due to their potential for scalability and cost-effectiveness. Traditional recombinant protein production, while offering precise control over therapeutic molecules, often requires complex and expensive purification processes that limit commercial viability in animal production. However, recent advances in protein engineering have revealed that certain protein aggregates, previously considered production waste, may offer unique advantages as delivery systems. This discovery has led to growing interest in inclusion bodies (IBs), a class of naturally derived biomaterials consisting of nanostructured recombinant protein aggregates that combine biological activity with inherent stability and self-delivery properties, potentially addressing both efficacy and economic constraints that have limited adoption of other antimicrobial alternatives in livestock and aquaculture (Pares et al., 2021; Lopez-Cano et al., 2022a; Torrealba et al., 2024).

**Fig. 1:**
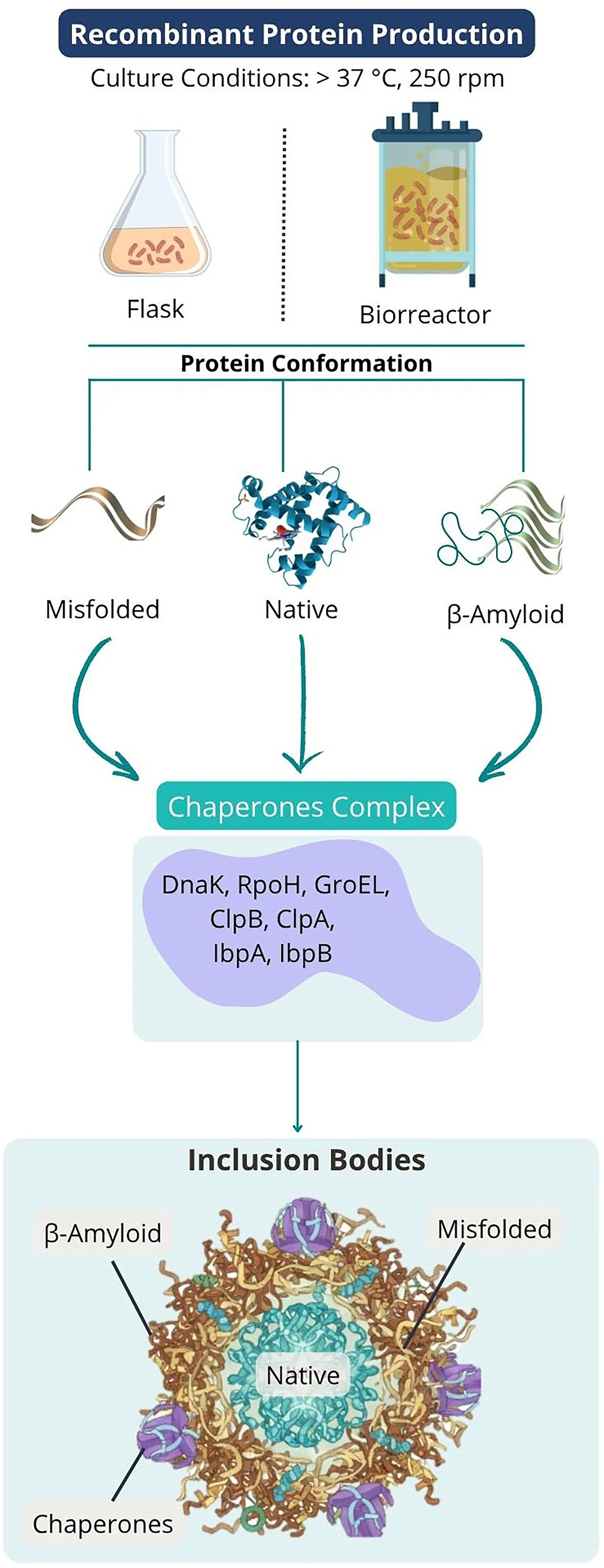
Formation and structure of bacterial inclusion bodies (IBs) as functional biomaterials.

IBs are produced naturally during heterologous protein expression (de Marco et al., 2019) in bacteria and yeast expression systems (Rueda et al., 2016) (Fig. 1). Although long considered waste by-products, research over the past decade has demonstrated that nanostructured recombinant proteins from IBs retain biological functionality (Vazquez et al., 2012). One of the main advantages of IBs is that they function as delivery systems for bioactive compounds, eliminating the cost of designing and producing additional delivery mechanisms. IBs possess β-amyloid structure that confers mechanical robustness and stability to the recombinant proteins forming them (de Groot et al., 2009). Approximately 40% of IBs consist of mechanically stable a β-amyloid fibrils, which surround and protect the remaining fraction, containing correctly folded protein. This protein can be released under physiological conditions and is responsible for the biological activity of IBs (Cano-Garrido et al., 2013). The combination of biological activity, mechanical stability, and high porosity makes these biomaterials an innovative and unconventional functional nanomaterial that can be tailored to package any functional polypeptide of interest (Slouka et al., 2019). Notably, IB production has evolved rapidly in recent years, incorporating small artificial peptides and large protein domains to promote efficient aggregation of soluble proteins into IBs regardless of culture conditions (Krauss et al., 2017; Jager et al., 2020).

IBs have been evaluated for disease control across various animal species, including fish such as sole and rainbow trout (Torrealba et al., 2016a; Thwaite et al., 2020); livestock including pigs, cattle, and sheep (Wedrychowicz et al., 2007; Pares et al., 2021; López-Cano et al., 2022); and model organisms such as zebrafish and mice (Rivera & Espino, 2016; Ji et al., 2019). IBs have demonstrated potential in controlling fish bacterial pathogens such as *Pseudomonas aeruginosa* (Torrealba et al., 2016a), which causes ulcerative syndrome and hemorrhagic septicemia (Algammal et al., 2020), and Spring viremia of carp virus (SVCV) (Rojas-Peña et al., 2022), a highly pathogenic virus that causes severe inflammatory responses in infected carp (Yu et al., 2025). In livestock, prototype vaccines based on IBs have been developed against *Lawsonia intracellularis* (Salazar et al., 2021), the causative agent of Porcine Proliferative Enteropathy (Rodriguez-Vega et al., 2024). Additionally, IBs containing cytokines have been evaluated as immunostimulants in pigs (López-Cano et al., 2022). Despite this potential, widespread adoption of this technology has not occurred, as no commercial products based on these biomaterials are currently available for aquaculture or livestock applications.

Despite growing research demonstrating the potential of IBs for disease control in animal production, no comprehensive systematic review has been conducted to synthesize and critically evaluate available evidence. Previous narrative reviews have addressed IB applications in broader biotechnological contexts (de Marco et al., 2019; Singhvi et al., 2020), but none have specifically focused on therapeutic applications in aquaculture and livestock using systematic methodology. The scattered nature of current research across different animal models, pathogens, and administration routes makes it challenging for researchers and industry stakeholders to assess the true potential and limitations of this technology. Furthermore, heterogeneity in study designs, evaluation metrics, and reporting standards has hindered translation of promising laboratory results into practical applications.

This systematic review addresses critical knowledge gaps by providing the first comprehensive synthesis of evidence regarding IB efficacy and practical applications for disease control in farmed animals. Specifically, this review aims to: (1) evaluate the types of proteins and expression systems used for IB production in animal health applications, (2) assess the effectiveness of different administration routes and dosing strategies, (3) analyze immunomodulatory effects and protective outcomes achieved with IB-based treatments, and (4) identify technological challenges that limit translation of IBs from laboratory to commercial applications in aquaculture and livestock industries. By systematically analyzing available literature according to PRISMA guidelines, this review establishes a robust evidence base to inform the development of IB-based interventions in animal health and guide future research directions.

## 2 Methods

### 2.1 Protocol and search strategy

**Fig. 2:**
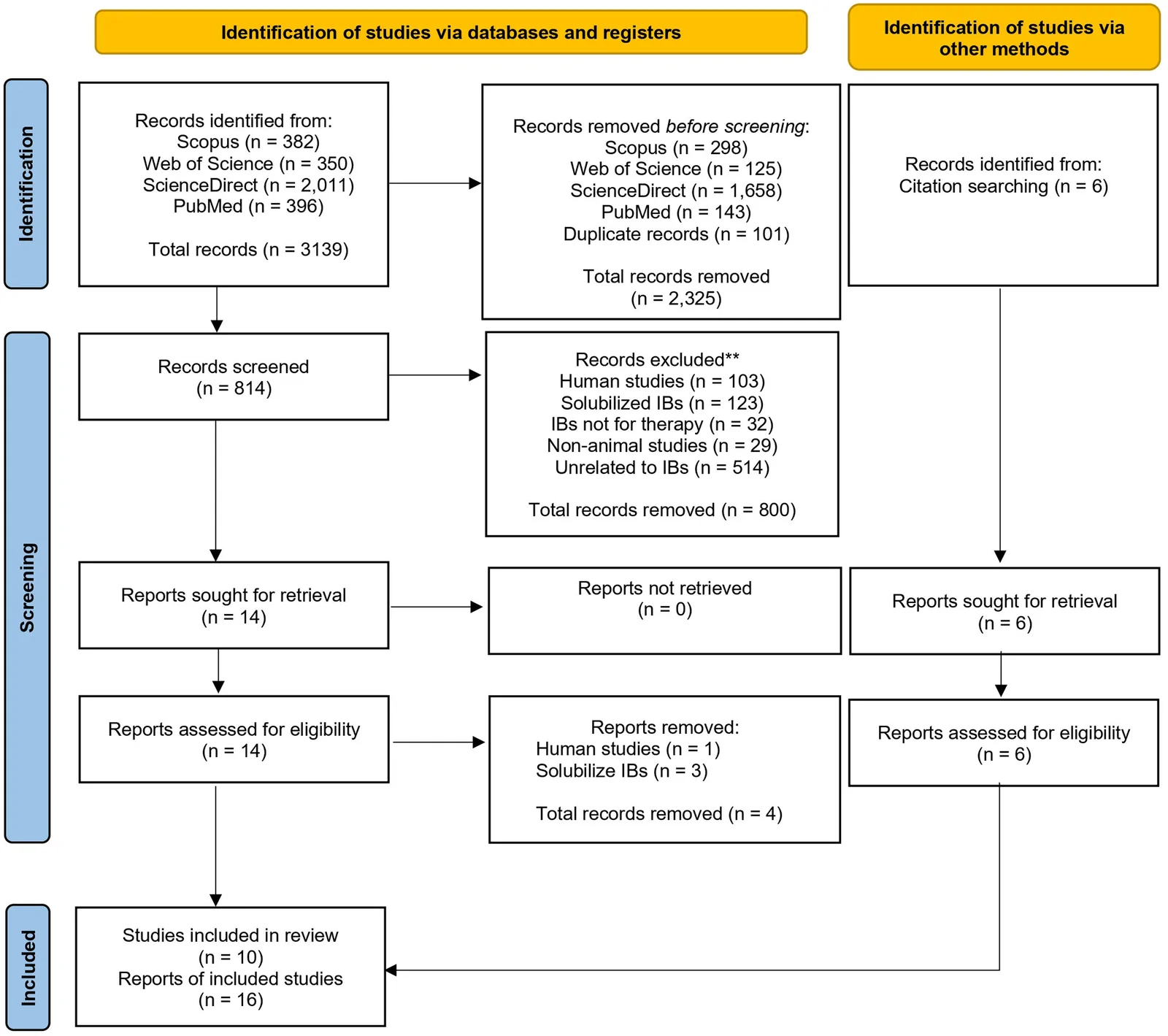
Flow diagram of the systematic literature search and study selection process (adjust from Page et al. (2021)).

This systematic review was conducted following Preferred Reporting Items for Systematic Reviews and Meta-Analyses (PRISMA) guidelines (Page et al., 2021) to ensure transparent reporting of the review process (Fig. 2). A comprehensive literature search was performed across four databases: Scopus, Web of Science, ScienceDirect, and PubMed. The search was limited to research articles in English published between 2000 and 2025, to capture relevant research following the initial development of inclusion body technologies.

Database-specific search strategies were developed using a combination of controlled vocabulary and free-text terms organized around three conceptual blocks: (1) IB identity terms ("inclusion bodies" OR "nanostructured protein"); (2) application terms (prophylactic OR therapies OR therapy OR immunostimulants OR immunomodulatory OR treatment); and (3) exclusion filters using NOT operators (AND NOT human AND NOT plant AND NOT child*ren AND NOT virus AND NOT patient AND NOT "sars COV-2"). All terms were adapted for each database to account for differences in indexing and syntax (Table 1).

**Table 1:** Search strategies and exclusion filters by database.

Database	Search Strategy	Date
PubMed	(("inclusion bodies" OR "nanostructured protein") AND (prophylactic OR therapy OR therapies OR immunostimulants OR immunomodulatory OR treatment) NOT human NOT plant NOT child*ren NOT virus NOT patient NOT "sars COV-2")	09/09/2025
Web of Science	(("inclusion bodies" OR "nanostructured protein") AND (zebrafish OR prophylactic OR therapies OR therapy OR immunostimulants OR immunomodulatory OR treatment) NOT human NOT plant NOT child*ren NOT virus NOT patient NOT "sars COV-2")	08/09/2025
ScienceDirect	(("inclusion bodies" OR "nanostructured protein") AND (therapy OR immunostimulants OR treatment) (NOT human NOT plant NOT virus))	06/09/2025
Scopus	((("inclusion bodies" OR "nanostructured protein") AND (zebrafish OR prophylactic OR therapies OR therapy OR immunostimulants OR immunomodulatory OR treatment) AND NOT human AND NOT plant AND NOT child*ren AND NOT virus AND NOT patient AND NOT "sars COV-2"))	06/09/2025

### 2.2 Eligibility criteria

Inclusion criteria (PICOS framework) were as follows: (1) Population: aquaculture species (fish) and livestock animals (cattle, pigs, sheep) at any developmental stage; (2) Intervention: administration of inclusion bodies containing recombinant proteins for disease prevention or treatment; (3) Comparator: control groups (untreated, placebo, or conventional treatments); (4) Outcomes: primary outcomes included immune response modulation, pathogen resistance, survival rates, and disease control efficacy; and (5) Study design: original research articles. Additional inclusion criteria comprised studies published in English, peer-reviewed journal articles, studies using intact (non-solubilized) inclusion bodies, and studies reporting quantitative or qualitative outcomes.

Exclusion criteria were human studies or in vitro studies without animal validation, studies focusing on solubilized IBs or purified proteins, review articles, conference abstracts, case reports, or editorials, studies not available in English, studies where IBs were not the primary therapeutic intervention, and plant-based studies or microbial applications without animal testing. Articles not related to inclusion bodies, that is, those examining signaling pathways, receptors, or the nature and structure of non-inclusion body molecules, were also excluded. Database filters were employed for article preselection (Table 1).

### 2.3 Study selection

All original scientific articles containing the mentioned terms in the title, abstract, or introduction were included for initial screening. Publications meeting inclusion criteria were entered into EndNote Online (Clarivate Analytics, London, United Kingdom) reference manager and organized by primary authors. Two independent reviewers screened all retrieved titles and abstracts using predefined eligibility criteria. Disagreements were resolved through discussion, with a third reviewer consulted when consensus could not be reached. Articles passing initial screening underwent full-text evaluation by the same two reviewers. Reasons for exclusion at this stage were documented.

### 2.4 Data extraction

Data extraction was performed independently by two reviewers using a standardized data extraction form developed specifically for this review. Discrepancies were resolved through discussion, with involvement of a third reviewer when necessary. The following data were systematically extracted from each included study: (1) author(s) and publication year; (2) study design and duration; and (3) sample size. Intervention details extracted included: (1) type of recombinant protein produced as biomaterial IBs; (2) expression system (bacterial strain, vector, and culture conditions); (3) purification and processing methods; and (4) protein characterization methods. Population characteristics recorded were: (1) animal species; (2) age or developmental stage; and (3) number of animals per group or experiment. Treatment protocols included: (1) administration route; and (2) dose and dosing regimen. Finally, innate immune parameters (e.g., cytokines), adaptive immune parameters (e.g., immunoglobulins), and Relative Percentage of Survival (RPS) were also extracted. Results were organized according to selected criteria and tabulated.

## 3 Results and discussion

The search strategy yielded 3139 documents after applying database filters (date range: 2000–2025; English language only) distributed as follows: 382 from Scopus, 350 from Web of Science, 2011 from ScienceDirect, and 396 from PubMed (Fig. 2). Following application of database-specific filters (Table 1), 2325 articles were excluded across databases: Scopus (n = 298), Web of Science (n = 125), ScienceDirect (n = 1658), and PubMed (n = 143), in addition to 101 duplicates. Subsequent title and abstract screening excluded a further 800 documents on the following grounds: unrelated inclusion bodies (n = 514), solubilization of inclusion bodies (n = 123), human studies (n = 103), inclusion bodies not for therapy (n = 32), and non-animal studies (n = 29), leaving 14 reports for full-text assessment. Of the remaining 14 full-text articles reviewed, 4 were excluded, one involving human subjects and three for focusing on solubilization of inclusion bodies, yielding 10 articles meeting inclusion criteria from database searches. Citation searching of included studies and relevant reviews identified 6 additional eligible studies, for a total of 16 studies included in this systematic review (Fig. 2).

### 3.1 IB production: proteins and expression systems

The recombinant proteins produced as IBs across all reviewed studies can be classified as either host-specific proteins (5 of 16 articles) or pathogen-specific proteins (11 of 16 articles) (Table 2). Among host-specific approaches, several immune system proteins have been explored for disease control. Proinflammatory cytokines, including tumor necrosis factor-alpha (TNFα), interleukin 1-beta (IL1β), and interleukin 6 (IL6), produced predominantly by activated macrophages, have been evaluated as immunostimulants due to their roles in inflammatory processes (Secombes, 2022). Chemokines have also been investigated, including interleukin 8 (IL8 or chemokine (C—C motif) ligand 8), a proinflammatory molecule (Zhu et al., 2020), and chemokine (C—C motif) ligand 4 (CCL4), a chemoattractant that recruits immune cells to sites of infection or tissue damage (Mackenzie et al., 2004). Beyond cytokines and chemokines, two studies explored the use of IB-based biomaterials containing matrix metalloproteinase-9 (MMP9) to trigger immune function (Gifre-Renom et al., 2020a; Pares et al., 2021). Additionally, IBs of antimicrobial peptides have been produced and evaluated for their antimicrobial and immune response-modulating capacity (Roca-Pinilla et al., 2020; Lopez-Cano et al., 2022b; Torrealba et al., 2024). Together, these biomaterials have demonstrated the ability to activate host immune responses, thereby enhancing immunity and improving animal health status before and during disease episodes.

**Table 2:** Recombinant proteins produced as IB-based biomaterials for disease control in aquaculture and livestock.

Scope of application	Types of protein	IBs Produced	Expression vector	Expression system	Reference
Livestock	Pathogen-specific	CPFhW	pIGDMCT7RS	*E. coli* BL21(DE3)	(Wedrychowicz et al., 2007)
Livestock	Pathogen-specific	OMP1c, OMP2c INVASc	pET22b(+)	*E. coli* Shuffle T7 competent	(Montesino et al., 2019)
Livestock	Pathogen-specific	OMP1c, OMP2c INVASc	pLawVac	*E. coli* Shuffle T7 competent	(Salazar et al., 2021)
Livestock	Pathogen-specific	OMP1c, OMP2c INVASc	pLawVac	*E. coli* Shuffle T7 competent	(Salazar et al., 2021)
Livestock	Pathogen-specific	OmpK-His, OmpK-GST, OmpK-MBP	pET22b(+)	*E. coli* DH5α and *E. coli* BL21	(Wang et al., 2021)
Livestock	Host-specific	MMP9	pNZ8148	*Lactococcus lactis* subsp. *cremoris* NZ9000	(Gifre-Renom et al., 2020a)
Livestock	Host-specific	MMP9	pNZ8148	*Lactococcus lactis* subsp. *cremoris* NZ9000	(Pares et al., 2021)
Livestock	Host-specific	IL1β, IL6, IL8, TNFα	pNZ8148	*Lactococcus lactis* subsp. *cremoris* NZ9000	(Lopez-Cano et al., 2022a)
Aquaculture	Pathogen-specific	VP1	pTVP1GFP	*E. coli* K-12 strains JGT4 and KPM335	(Torrealba et al., 2016b)
Aquaculture	Pathogen-specific	CP-VNNV, GP-VHSV CP-IPNV	pET22b(+)	*E. coli* BL21(DE3)	(Thwaite et al., 2018)
Aquaculture	Pathogen-specific	GP-SVCV, GP-SVCV-IFN	pET22b(+)	*E. coli* BL21(DE3)	(Rojas-Pena et al., 2022)
Aquaculture	Pathogen-specific	CP-VNNV	pET22b(+)	*E. coli* BL21(DE3)	(Thwaite et al., 2020)
Aquaculture	Host-specific	TNFα	pQE30	*E. coli* M15	(Ji et al., 2019)
Aquaculture	Host-specific	CCL4, TNFα	pET30Xa/LIC pQE30	*E. coli* DH5α *E. coli* M15[pREP4]	(Torrealba et al., 2016a)
Livestock	Pathogen-specific	FhSAP2	pBAD	E. coli TOP10	(Rivera & Espino, 2016)
Livestock	Pathogen-specific	ETEC-FaeG-mSTa-LTb-STb	pProEXHT	*E. coli* BL21	(Jiang et al., 2018)

**CCL4:** Chemokine (C—C motif) ligand 4; **CP-VNNV:** Coat protein from the Iberian betanodavirus; **CP-IPNV:** Capsid protein 2 from the Infectious pancreatic necrosis virus; **CPFhW:** Cysteine proteinase of F. hepatica; **ETEC:** Enterotoxigenic *E. coli*; **FaeG:** fimbrial adhesin subunit protein; **FhSAP2**: saposin-2 of *Fasciola hepatica*; **GP-SVCV:** Glycoprotein G of Spring viremia of carp virus; **GP-SVCV-IFN:** Glycoprotein G of Spring viremia of carp virus with an interferon gamma module; **GP-VHSV:** Glycoprotein G from the viral hemorrhagic septicemia virus; **IFNγ:** Interferon gamma; **IL1β:** Interleukin 1 beta; **IL6:** Interleukin 6; **IL8:** Interleukin 8; **INVASc:** Invasin (L. intracellularis secretion protein); **MMP9:** matrix metalloproteinase-9; **Lts:** heat-labile enterotoxins; **OMP1c/OMP2c:** L. intracellularis outer membrane protein 1 or 2; **OmpK:** Vibrio sp outer membrane protein K; **OmpK-GST:** OmpK fusion tag glutathione S-transferase; **OmpK-MBP:** OmpK fusion maltose binding protein; **STs:** heat-stable enterotoxins; **TNFα:** Tumor necrosis factor alpha; and **VP1:** protein of the capsid of the foot-and-mouth disease virus.

Studies on pathogen-specific proteins aim to activate adaptive immune cell memory, functioning similarly to vaccines. In this context, these biomaterials have been produced with antigenic proteins from various pathogens, including bacteria such as *Lawsonia intracellularis* and *Vibrio sp.*; viruses such as Viral nervous necrosis virus (VNNV), viral hemorrhagic septicemia virus (VHSV), and spring viremia of carp virus (SVCV); as well as the parasite *Fasciola hepatica* (Table 2). For viral targets, viral capsid proteins have been explored for IB production, as this subunit vaccine format provides a safe alternative with no risk of host DNA integration, reversion, or invasion. For bacterial targets, highly antigenic outer membrane proteins (Omp) have proven to be excellent immune response activators (Rainard et al., 2017; Harshitha et al., 2023).

Among known expression systems for recombinant protein production, this review identified two expression systems used for IB production in animal applications: *Escherichia coli* and *Lactococcus lactis subsp. cremoris* (Table 2). *L. lactis* was preferred in livestock studies, likely due to its Generally Recognized as Safe (GRAS) status and lack of endotoxin production (Singh & Singh, 2022). In contrast, *E. coli* is widely used due to its high expression yields, although its lipopolysaccharides (LPS) can trigger strong proinflammatory responses in mammals. Notably, teleost fish exhibit greater LPS tolerance due to low TLR4-mediated sensitivity (Martinez-Lopez et al., 2023), a property that has been exploited to explore LPS as an immunostimulant in aquaculture species such as rainbow trout and Atlantic salmon (Nya & Austin, 2010; Sahoo et al., 2017).

### 3.2 Delivery routes and biodistribution

Several routes of IB administration have been studied, including intramuscular and subcutaneous injection, nasal suspension, and mammary infusion in livestock, as well as intraperitoneal administration and bath immersion in aquaculture (Table 3). Oral administration has also been explored in both aquaculture and livestock, as it represents the simplest, most cost-effective, and least stressful method, particularly in fish farming, where large numbers of individuals can be immunized with minimal resources (Mutoloki et al., 2015).

**Table 3:** Disease targets, animal models, and administration strategies of IB-based biomaterial for disease control in livestock and aquaculture.

Organism	Stages of development	Disease or pathogens target of IBs	Administration strategy	Doses	Reference
*Bos taurus*	adult	mastitis	intra-Mammary gland infusion	0.012 to 12 mg	(Gifre-Renom et al., 2020a)
*Bos taurus*	adult	mastitis	intra-mammary gland infusion	12 mg	(Pares et al., 2021)
*Bos taurus*	calve	*Fasciola hepatica*	nasal suspension	500 μg	(Wedrychowicz et al., 2007)
*Ovis aries*	lamb	*Fasciola hepatica*	nasal suspension, gastric injection, esophageal intubation, intramuscular injection	300 μg	(Wedrychowicz et al., 2007)
*Sus domesticus*	piglet	N/R	oral	20 µg/kg	(Lopez-Cano et al., 2022a)
*Sus domesticus*	piglet	*Lawsonia intracellularis*	intramuscular injection	200 μg/mL	(Salazar et al., 2021)
*Sus domesticus*	piglet	*Lawsonia intracellularis*	intramuscular injection	100 μg/mL, 200 μg/mL	(Salazar et al., 2021)
*Sus domesticus*	piglet	*Lawsonia intracellularis*	intramuscular injection	200 μg/mL	(Montesino et al., 2019)
*M. musculus*	adult	*Lawsonia intracellularis*	intramuscular injection	12.5 μg/mL	(Salazar et al., 2021)
*M. musculus*	adult	*Lawsonia intracellularis*	intramuscular injection	5 μg/mL	(Montesino et al., 2019)
*M. musculus*	adult	*Vibrio* sp.	subcutaneous injection	80 μg/mL, 40 μg/mL	(Wang et al., 2021)
*Oncorhynchus mykiss*	adult	N/R	oral intubation	7.5 mg/kg	(Torrealba et al., 2016a)
*Solea senegalensis*	juvenile	Nervous necrosis virus	peritoneal injection, oral intubation	50 μg/fish, 500 μg/fish	(Thwaite et al., 2020)
*Danio rerio*	adult	*Pseudomonas aeruginosa*	peritoneal injection	5.5mg/kg	(Torrealba et al., 2016a)
*Danio rerio*	adult	*Pseudomonas aeruginosa*	peritoneal injection	300 μg/mL, 150 μg/mL, 75 μg/mL, 50 μg/mL, 25 μg/mL	(Torrealba et al., 2016b)
*Danio rerio*	larvae	*Mycobacterium marinum*	bath immersion	10 25 μg/mL, 50 μg/mL	(Ji et al., 2019)
*Danio rerio*	adult	*Mycobacterium marinum*	oral intubation	10 μg/fish, 20 μg/fish	(Ji et al., 2019)
*Danio rerio*	adult	*Mycobacterium marinum*	peritoneal injection	10 μg/fish, 20 μg/fish	(Ji et al., 2019)
*Danio rerio*	larvae	N/R	bath immersion	10 µg/mL	(Rojas-Pena et al., 2022)
*Danio rerio*	adult	Spring viremia of carp virus	peritoneal injection	5 µg /fish	(Rojas-Pena et al., 2022)
*M. musculus*	adult	*Fasciola hepatica*	subcutaneous injection	20 μg	(Rivera & Espino, 2016)
*Danio rerio*	adult	N/R	oral intubation	20 μg/fish	(Thwaite et al., 2018)
*M. musculus*	adult	ETEC	oral	50 µg	(Jiang et al., 2018)

N/R: Not Reported.

The remarkable stability of these biomaterials under various physicochemical conditions, including lyophilization, high temperatures, and pH variations (Torrealba et al., 2016a; Rojas-Pena et al., 2022), is a key advantage for their practical application. For lyophilization, IBs were incubated at −80 °C for 8 h and subsequently stored for three weeks at room temperature (Torrealba et al., 2016a). Under these conditions, IBs maintained size and morphology while retaining protective efficacy in zebrafish challenged with lethal bacteria, with no significant differences compared to non-lyophilized IB controls, confirming that bioactivity is preserved despite lyophilization.

Commercial fish food production involves extrusion processes where ingredient mixtures are subjected to high temperatures for brief periods to achieve continuous and uniform pellet formation. When these biomaterials were exposed to similar high-temperature conditions (100 °C for 30 s or 5 min), field emission scanning electron microscopy (FESEM) analysis revealed no changes in shape or size (Torrealba et al., 2016a; Rojas-Peña et al., 2022). Comparable results were observed when IBs were incubated under pH conditions mimicking gastrointestinal environments of fish: sequential exposure to pH 2.5 for 3 h followed by pH 8 for 6 h, or independent incubation at pH 2 and pH 10 for 3 h each (Torrealba et al., 2016a; Rojas-Peña et al., 2022). However, these assessments were limited to morphological characterization, confirming absence of IB degradation. Additional studies are required to deeply evaluate IB bioactivity following exposure to these harsh conditions. Collectively, these stability characteristics, which typically present significant challenges for soluble recombinant proteins, make IB-based biomaterials particularly advantageous for oral administration, as their implementation could substantially reduce animal handling and associated stress, both critical factors in maintaining immune function and overall health.

Livestock studies have demonstrated that these biomaterials induce immune responses regardless of administration route. Whether administered orally, intramuscularly, or via infusion, IBs have consistently triggered measurable immunological effects. This suggests that differences in delivery methods do not compromise efficacy but rather offer flexible strategies to adapt to the physiological characteristics of each species and specific needs of disease management. However, oral administration in ruminants presents unique challenges, as antigens must pass through four stomach compartments before reaching the intestine to elicit immune responses (Wedrychowicz et al., 2007). Despite these challenges, in pigs fed IB-IL1β at 20 µg/kg, IB-TNFα levels significantly increased at 7 days post-treatment, although no significant difference in blood cytokines was observed at 24 h compared to controls (Lopez-Cano et al., 2022a). Similarly, intramuscularly administered IBs have enhanced both humoral and cellular immune markers, reinforcing their potential as immunostimulants (Salazar et al., 2023). Comparable results were observed following mammary gland infusion of MMP-9 IBs in cows, where enzymatic activity peaked between the third and sixth-day post-administration (Pares et al., 2021). Other biomarkers, such as BSA and Na+/K+ ratios, also exhibited increased levels around the sixth day, further supporting sustained immune responses triggered by IB administration (Gifre-Renom et al., 2020a). Taken together, these findings suggest that IB-based biomaterial consistently activate immune responses irrespective of administration route, as evidenced by significant upregulation of immune biomarkers across species and delivery strategies.

In fish, these biomaterials have been demonstrated that after intubation administration, IBs are absorbed by intestinal cells in *Oncorhynchus mykiss* and subsequently reach key immune organs such as head kidney and spleen, representing a significant breakthrough in IB delivery strategies (Torrealba et al., 2016a). Similar findings have been reported for zebrafish, where IBs administered via immersion bath were internalized within 24 h, suggesting efficient uptake mechanisms (Thwaite et al., 2018; Ji et al., 2019). This uptake triggers innate immune responses at mucosal barriers through epithelial and antigen-presenting cells, leading to activation of B and T lymphocytes, cytotoxic T cell responses, and antibody production (Kunisawa & Kiyono, 2005), reinforcing the importance of mucosal delivery routes. Additionally, intraperitoneal administration has demonstrated high effectiveness, significantly enhancing both innate and adaptive immune markers, further supporting its potential as a powerful strategy for IB-based immunization (Rojas-Peña et al., 2022).

Despite their versatility across delivery methods, IB-based antigen and cytokine treatments exhibit strong immunostimulatory activity. For instance, while oral administration is convenient and minimally stressful, it may be unsuitable for ruminants due to their complex gastrointestinal anatomy, which can degrade antigens before they reach the intestine. Similarly, in aquaculture, although immersion and oral administration are practical options for mass immunization, they present challenges. Both methods show inconsistent results, require constant reinforcement that raises final costs, and are vulnerable to unpredictable feeding behavior that can undermine their adoption (Tammas et al., 2024). Conversely, intramuscular or subcutaneous injections provide more direct and controlled antigen delivery but require increased handling, which can elevate stress levels and potentially impact immune efficacy, particularly in aquaculture. Given these variations, future research should prioritize the optimization of biomaterial administration strategies by considering species-specific physiological and immunological traits, the characteristics of the target pathogens, and the animal farming conditions. Deeper understanding of these factors will be essential for maximizing the therapeutic potential of these biomaterials and ensuring their successful application in both aquaculture and livestock.

### 3.3 Immunomodulatory potential of IB-based biomaterials

The disease control applications of IBs encompass multiple pathogenic targets across aquaculture and livestock production systems (Table 2). In aquaculture, pathogens include *Pseudomonas aeruginosa, Mycobacterium marinum*, Spring viraemia of carp virus (SVCV), and Viral nervous necrosis virus (VNNV), with significant impacts on freshwater and Mediterranean aquaculture. In livestock, diseases and pathogens evaluated include mastitis, *Fasciola hepatica, Lawsonia intracellularis*, and *Vibrio sp*., affecting various domestic species*.* In these studies, the disease control potential of IBs centers on two mechanisms: modulation of the immune system when host immune proteins are produced, and activation of immune responses when pathogen-derived antigenic proteins are produced.

Generally, evaluation of immune responses to IBs is assessed by analyzing relative expression of genes and abundance of proteins associated with innate or adaptive immune responses (Table 4). Cytokines are biomarkers commonly used in this assessment (Sakai et al., 2020; Vitenberga-Verza et al., 2022). Studies show trends toward increased gene expression related to proinflammatory cytokines such as *tnfα, il1β*, and interleukin 12 (*il12*) following administration of IBs with proinflammatory potential (Table 4). Additionally, clear modulation of the immune responses is evident in some studies. For example, *in vitro* assays with IB-IL1β show significant stimulation of *tnfα* gene expression in epithelial cells but not in macrophages within 16 h (López-Cano et al., 2022). *In vivo* assays in pigs show tendencies for *tnfα* gene expression to increase 7 days after treatment (López-Cano et al., 2022). In fish, IB-IL1β is related to *tnfα* gene overexpression but not *il6*, whereas IB-TNFα stimulates expression of *tnfα, il6, il1β*, and *il8* (Torrealba et al., 2016a). Each protein is related to increased expression of specific gene sets for the model studied. Interestingly, the same IB-TNFα protein does not significantly increase *tnfα* gene expression in the intestine and spleen of *Danio rerio*, but does regulate overexpression of *il1β* and *il6* in these tissues (Ji et al., 2019). Overall, these findings indicate that each IB-based biomaterial formulation elicits distinct gene expression profiles, underscoring the importance of tailoring treatments to specific immune characteristics of target models (Table 5).

**Table 4:** Innate immune parameters modulated by IBs-based biomaterials in reviewed studies.

IBs Treatment	Marker type		Reference
	Gene	Protein	
Pathogen-specific: CP-VNNV, CP-IPNV, GP-VHSV	↑*vig1,* ↑*gig2,* ↑*stat1b,* ↑*mx,* ↑*irf7,* ↑*ccl4*	N/R	(Thwaite et al., 2018)
Pathogen-specific: OMP1c, OMP2c, INVASc	↑*il12,* ↑*oas2,*	N/R	(Montesino et al., 2019)
Pathogen-specific: GP-SVCV, GP-SVCV-IFN	*↑tnfα, ↑il1β, ↑il10, ↑vig1, ↑mx, ↑lmp2, ↑irf1, ↑ccl4, ↑ifngr1, ↑prf19b, ↑gzma*	N/R	(Rojas-Pena et al., 2022)
Host-specific: CCL4, TNFα	↑*tnfα,* (n.s.) *il6,* ↑*il1β,* ↑*il8,* ↑*cox2,* (n.s.) *camp1,* (n.s.) *socs3,* ↑*mmp9*	N/R	(Torrealba et al., 2016a)
Host-specific: TNFα	↑*il1β,* ↑*il6,* ↑*il10,* (n.s*.*) *il22,* ↑*cox2,* (n.s.) *c3,* ↑*mmp9,* ↑*tlr5*	N/R	(Ji et al., 2019)
Host-specific: MMP9	N/R	↑Lactoferrin, ↑BSA	(Pares et al., 2021)
Host-specific: MMP9	N/R	↑Lactoferrin, ↑BSA	(Gifre-Renom et al., 2020a)
Host-specific IL1β, IL6, IL8, TNFα	(n.s.) *tnfα,* (n.s.) *cldn4,* (n.s.) *bd1,* (n.s.) *bd2,* (n.s.) *il6*	(n.s.) IL8, (n.s.) IL10, (n.s.) IL6, (n.s.) TNFα	(Lopez-Cano et al., 2022a)
Pathogen-specific: FhSAP2	N/R	(n.s) IFNγ, ↓IL-4, ↑TNFα	(Rivera & Espino, 2016)

**bd1:** b-defensin-1; **bd2:** b-defensin-2; **BSA:** bovine serum albumin; **c3:** complement c3; **camp1:** cathelicidin antimicrobial peptide; **ccl4:** c-c motif chemokine ligand four; **cldn4:** claudin four; **cox2:** cyclooxygenase-2; **gig2:** grass carp reovirus induce gene two; **gzma:** granzyme A; **ifngr1:** interferon gamma receptor one; **il1*β*:** interleukin one beta; **il6:** interleukin 6; **il8:** interleukin eight; **il10:** interleukin ten; **il12:** interleukin twelve; **il22:** interleukin twenty two; **irf1:** interferon one; **irf7:** interferon seven; **lmp2:** latent Membrane Protein 2; **mmp9:** matrix metalloproteinase-9; **mx:** myxovirus resistance; **n.s.:** non-significant difference with respect to the control; **N/R:** not reported; **oas2:** 2′5′-oligoadenylate synthetase 2; **prf19b:** pre-mRNA processing factor 19; **socs3:** suppressor of cytokine signaling 3; **stat1b:** signal transducer and activator of transcription 1; **tlr5:** toll-like receptor five; **tnfα:** tumor necrosis factor alpha; **vig1:** virus-induce gen 1.

**Table 5:** Adaptive immune parameters modulated by IB-based biomaterials in reviewed studies.

IBs Treatment	Marker type		Reference
	Gene	Protein	
Pathogen-specific: CPFhW	N/R	*↑*GGT, *↑*IgG, ↓IgA	(Wedrychowicz et al., 2007)
Pathogen-specific: OMP1c, OMP2c, INVASc	(n.s.) *cd4*	*↑*CD4^+^, *↑*IgG	(Montesino et al., 2019)
Pathogen-specific:CP-VNNV	↑*arg2* (intestine)*,* ↑*arg2* (spleen), *↓igt, ↓cd8a,* ↑*cd4,* ↑*igm,* ↑*cd8*	*↑*IgM	(Thwaite et al., 2020)
Pathogen-specific: OMP1c, OMP2c, INVASc	N/R	↑IgG	(Salazar et al., 2021)
Pathogen-specific: OMP1c, OMP2c, INVASc	↑*ifnγ,* ↑*il12,* ↑*il4*	↑IFNγ, ↑IgG	(Salazar et al., 2021)
Pathogen-specific: OmpK-GST OmpK-MBP	N/R	↑IgG	(Wang et al., 2021)
Pathogen-specific: FhSAP2	N/R	↑IgG1, ↑IgG2a	(Rivera & Espino, 2016)
Pathogen-specific: ETEC-FaeG-mSTa-LTb-STb	N/R	↑IgG2b, ↑IgA, ↑IgM,	(Jiang et al., 2018)

**arg2:** arginase; **cd4:** cluster of differentiation 4; **cd8a:** cluster of differentiation 8 alpha; **GGT:** gamma-glutamyltransferase; **ifnγ:** interferon gamma; **IgA:** immunoglobulin A; **IgG:** immunoglobulin G; **IgM:** immunoglobulin M; **igt:** immunoglobulins T; **il4:** interleukin 4; **il12:** interleukin 12; **n.s.:** non-significant difference with respect to the control; **N/R:** not reported.

Therapeutic effects achieved with IBs largely depend on the design approach. IBs with antigenic properties can be co-formulated with proinflammatory cytokine-based adjuvants, such as SVCV-IFN, a chimeric protein designed to enhance interferon effects, leading to upregulation of innate immune genes (Rojas-Peña et al., 2022). Moreover, IBs have been shown to elicit enhanced adaptive responses in both fish and pigs by effectively stimulating innate immune systems (Montesino et al., 2019; Rojas-Peña et al., 2022). For instance, the expression of proinflammatory cytokines such as IL12 induces proliferation and differentiation of CD4⁺ T cells into Th1 cells, playing a critical role in their trafficking and migration (Hamza et al., 2010; Montesino et al., 2019). These observations indicate that the innate and adaptive immune systems act synergistically, orchestrated by the differential gene expression induced by recombinant protein treatments. Ultimately, this synergy reinforces the notion that the therapeutic outcomes of IB-based biomaterials are dependent on formulation design rather than delivery strategy, as the design approach determines how effectively these immune pathways are activated.

Long-term immunomodulatory effects of IBs have been evidenced in various animal models, demonstrating their capacity to sustain immune responses over time. For instance, Wedrychowicz et al. (2007) reported hematological changes in calves following administration of pathogen-specific antigen IBs, noting reduced eosinophilia (Wedrychowicz et al., 2007). Peak eosinophil activity occurred earlier in treated animals (week 4 in females and week 10 in males) compared to controls (weeks 8 and 14, respectively), suggesting that treatment with IBs accelerated eosinophil activation, potentially indicating faster and more efficient immune responses against specific pathogens or antigens used in this study. Similarly, Montesino et al. (2019) observed delayed but significant increases in IgG levels in C57BL/6 mice immunized with chimeric antigen IBs. While IgG levels remained low during the first two weeks post-immunization, a marked rise was detected by week 3, with immunized mice showing significantly higher levels than controls. These results confirm that IB-based biomaterials can activate immune systems and generate specific antibody responses, although the response requires several weeks to develop fully. These findings demonstrate the ability of these biomaterials to stimulate local immune cell recruitment over time, likely due to the controlled and prolonged release of soluble protein, which enhances antigen availability and immune system activation (Cespedes et al., 2016; Seras-Franzoso et al., 2016; Gifre-Renom et al., 2020b).

Although controlled release mechanisms of these biomaterials enhance antigen availability, there is currently no conclusive evidence demonstrating that booster doses are entirely unnecessary. Furthermore, detailed quantification of gradual release of antigens from IBs over time remains lacking, which raises questions about consistency and predictability of this mechanism. Without a clear understanding of antigen release kinetics, it is difficult to determine optimal dosing intervals or ensure that immune responses remain robust and sustained over extended periods. Variability in antigen release rates across different individual animals may further complicate practical application of these biomaterials in real-world settings. While the ability of IB-based biomaterials to stimulate both innate and adaptive immune responses positions them as promising platforms for next-generation vaccines and immunotherapies, further research is needed to fully characterize their release profiles and long-term efficacy. Addressing these gaps will be crucial for maximizing the potential of these biomaterials as reliable and effective tools for disease control in livestock and aquaculture.

Given that these biomaterials induce local and transient inflammatory responses, they often require repeated doses in some animal models when used as antigens (Wedrychowicz et al., 2007). This raises an important question: what is the necessary dose of IBs to induce a beneficial immune response, and for how long? The answer is that there is no linear relationship between dose and immune gene expression (Torrealba et al., 2016a); rather, expression of each gene varies depending on the treatment. For example, IB-TNFα at 20 µg and 5 µg significantly increases the cathelicidin antimicrobial peptide (*camp1*) gene expression, but there is no overexpression of this gene at a dose of 10 µg. In contrast, cyclooxygenase-2 (*cox2)* gene expression correlates with dose, being significantly higher at 20 µg and lower at 5 µg, while the *mmp9* gene expression is better at 5 µg (Torrealba et al., 2016a). Additionally, genes are expressed differentially over time. For instance, treatment with IB-TNFα overexpresses *il1β* and *cox2* genes for 5 h, but no significant expression of these genes is observed at 24 h, while *mmp9* and *il6* are significantly expressed (Ji et al., 2019)*.* However, this response is not immediate but requires several weeks to develop. These findings demonstrate the ability of IBs to stimulate local immune cell recruitment over time, likely due to the controlled and prolonged release of soluble protein, which enhances antigen availability and immune system activation. Collectively, these findings underscore the complexity of immune modulation by IB-based biomaterials, where both dose and timing play critical roles in shaping immune responses.

Innate immune responses are activated by both host immunostimulatory protein IBs and antigenic proteins, primarily mediated by neutrophils and receptor stimulation. These membrane receptors modulate immune-related molecules, enhancing both humoral and cellular responses (Thwaite et al., 2018; Gifre-Renom et al., 2020a). By triggering expression of inflammatory genes, these biomaterials provide a powerful tool to improve the immune performance of animals. However, effects of IB-based biomaterials can vary depending on animal model and, in some cases, sex. For example, female bovine models vaccinated with *F. hepatica* antigens show decreased parasite counts compared to males, indicating higher degrees of protection in females. Interestingly, parasites recovered in males were immature and smaller in size, suggesting a partial but less effective immune response (Wedrychowicz et al., 2007). In contrast, no sex-related differences in protection were observed in lambs, although overall protection was lower than in cattle (Wedrychowicz et al., 2007). These variations underscore the importance of considering species-specific and sex-related factors when designing IB-based therapies.

### 3.4 Protective efficacy: pathogen challenge and survival

Survival rates in pathogen challenges are critical factors when evaluating treatment effectiveness, as they directly reflect the ability to reduce mortality, improve animal health, and ensure economic profitability. Only 6 of 16 articles evaluated effects of these biomaterials on survival rates in fish against pathogen challenges (Table 6). All studies used RPS as survival measurement and employed zebrafish as the fish model with all treatments delivered intraperitoneally. Protective effect of different IBs against pathogens such as *P. aeruginosa, M. marinum*, and SVCV were demonstrated. However, the protection varied depending on the pathogen and nature of the protein used as treatment. For example, IB-TNFα shows an RPS of 100% for *P. aeruginosa i*nfection, while for *M. marinum* it is 33.3% at 30 days (Torrealba et al., 2016a; Ji et al., 2019). As with other classical vaccines, the addition of adjuvants to antigenic protein treatments contributes to increased RPS. For example, the antigenic protein IB-SVCV has an RPS of 50% without adjuvant and increases to 80% with the addition of IFN at 40 days (Rojas-Peña et al., 2022). Notably, the protective effect was sufficiently robust to persist even after lyophilization (Torrealba et al., 2016a). For example, in a challenge with *P. aeruginosa*, it was shown that RPS was higher for animals injected with lyophilized IB-TNFα (RPS 83%) than for non-lyophilized IB-TNFα (RPS 62%). Studies in *Mus musculus* have also demonstrated 80% survival following oral or intramuscular injection administration. Overall, these findings indicate that IB-based biomaterials have the potential to trigger a robust immune response capable of protecting animals against lethal pathogens, although further studies in commercially relevant species are needed to validate efficacy in real aquaculture settings.

**Table 6:** Relative Percent Survival (RPS) in animals treated with IB-based biomaterials following pathogen challenge.

Animal model	Target condition	Administration route	RPS (%)	Number of animals per treatment	Reference
*Danio rerio*	*Pseudomonas aeruginosa*	intraperitoneally	100 to 37	15	(Torrealba et al., 2016a)
*Danio rerio*	*Pseudomonas aeruginosa*	intraperitoneally	79 to 67	15	(Torrealba et al., 2016b)
*Danio rerio*	*Mycobacterium* *marinum*	intraperitoneally	33	12	(Ji et al., 2019)
*Danio rerio*	Spring viremia of carp virus	intraperitoneally	50 to 80	12	(Rojas-Pena et al., 2022)
*M. musculus*	ETEC	oral	80	15	(Jiang et al., 2018)
*M. musculus*	*Fasciola hepatica*	subcutaneous injection	80	10	(Rivera & Espino, 2016)

### 3.5 Technological challenges

The use of IB-based biomaterials as therapeutic approaches in animal production represents a promising strategy, particularly in vaccine development and controlled drug delivery systems. However, their implementation faces significant technological challenges that must be overcome to ensure effectiveness and safety. One of the main obstacles is the purification of target proteins. Currently, no standardized protocols guarantee complete purification of IBs, leading to the presence of bacterial residues in final products. In expression systems based on Gram-negative bacteria, these residues may include LPS, endotoxins that trigger severe inflammatory responses in mammals, such as high fever, tissue damage, and even death (Erridge et al., 2022). This poses major concerns when using these biomaterials for therapeutic purposes in livestock animals (Rodríguez-Carmona et al., 2010).

Although various procedures have been developed to remove LPS from recombinant protein preparations, achieving products with different purity levels (Liu et al., 1997; Petsch & Anspach, 2000; Magalhães et al., 2007), established approaches include the use of LPS-free expression systems such as *E. coli* mutant strains, lactic acid bacteria, or yeast (Rueda et al., 2016; Pares et al., 2021; Cardoso et al., 2022). More recently, an emerging strategy has gained attention: the *in vitro* assembly of purified proteins into synthetic IB-like nanostructures, also referred to as artificial inclusion bodies (Chen et al., 2020; Sanchez et al., 2020; Serna et al., 2023). These biomimetic materials retain the depot and controlled-release properties of naturally produced IBs while circumventing endotoxin contamination entirely. Proof-of-concept studies have already demonstrated their applicability for the delivery of antimicrobial peptides in zebrafish (Serna et al., 2023) and tumor-targeted fusion proteins in mice (Sanchez et al., 2020) and a vaccine platform in a hamster model (Parladé et al., 2025), although these applications have been primarily developed for human therapeutic purposes and their adaptation to animal production contexts remains to be explored. However, the translation of this technology to aquaculture and livestock settings warrants careful evaluation, as the additional purification and in vitro assembly steps required for artificial IB production may substantially increase manufacturing costs, a critical factor limiting the accessibility of vaccines and immunostimulants for cost-sensitive animal production industries (Rueda et al., 2014). In this context, even with lower-risk expression systems, such as Gram-positive bacteria, purification processes must be standardized to ensure final product quality and safety. Key advantages of Gram-positive systems such as *L. lactis* or *Lactiplantibacillus plantarum* (Lopez-Cano et al., 2022a; Balta-Foix et al., 2023) include their LPS-free nature and capacity to produce IBs with high purity, making them attractive alternatives to *E. coli*.

In recent years, preference has been given to purification approaches that combine physical and chemical treatments to enhance purification efficiency (Ferrer-Miralles et al., 2022). However, many of these methods are cost-effective and fast at laboratory scale but not feasible for industrial applications. The selection of appropriate methods should be based on a thorough understanding of the protein’s intrinsic characteristics, intended applications, and production processes (Singhvi et al., 2020). Overall, these factors hinder the transition of these biomaterials from laboratory research to practical application in the livestock industry, where profitability and efficiency are critical.

Another major technological challenge lies in the controlled production of these biomaterials. Although IB production processes are compatible with well-established recombinant protein production techniques, aggregation of these nanostructures is governed by physicochemical and structural characteristics of proteins, such as molecular weight, number of hydrophobic residues, and low-complexity regions (Dyson et al., 2004; Goh et al., 2004). Additionally, environmental conditions such as temperature and pH significantly influence the formation and stability of IBs. For instance, IBs formed at lower temperatures tend to solubilize more rapidly than those produced at higher temperatures, suggesting that cultivation temperature affects conformational properties of proteins within aggregates (de Groot & Ventura, 2006). Similarly, pH influences the tendency of β-conformations to form amyloid deposits (Sahdev et al., 2008). Consequently, the final characteristics of these biomaterials may vary significantly depending on the intrinsic properties of each protein and external production conditions, even among batches produced under seemingly controlled conditions. This variability threatens the efficacy and consistency of treatments, posing critical challenges for their application in animal production. Therefore, it is essential to develop optimized, case-specific strategies to maximize yield and ensure the capacity to meet industry demand for large-scale, cost-effective treatments. Achieving this balance between quality, efficiency, and scalability, through fine-tuning of parameters such as temperature, pH, and cultivation conditions, remains one of the biggest challenges in successfully implementing IB-based biomaterials in animal production.

Technological advances driven by artificial intelligence and computational biology are revolutionizing design and production of recombinant proteins. These systems can be adapted to enhance protein aggregate design, enabling molecular interactions that promote the formation of functional IBs. Advanced tools such as AlphaFold (Jumper et al., 2021; Pak et al., 2023), RoseTTAFold (Baek et al., 2021), ProtGPT2 (Ferruz et al., 2022), and machine learning models (Chowdhury et al., 2022) facilitate the prediction of protein sequences that, in the future, could be used to predict aggregation domains, thereby optimizing IB formation while preserving bioactivity. Furthermore, sophisticated algorithms can analyze big data from cultivation conditions to maximize both yield and purity, while automated platforms could accelerate strain selection and formulation optimization. These innovations will also enable the development of customized biomaterials featuring sustained-release properties and enhanced immunogenicity, positioning engineered IB-based biomaterials as scalable, cost-effective solutions for disease control in aquaculture and livestock.

### 3.6 Future perspectives

Recombinant protein markets were valued at USD 457.1 million in 2022 and are projected to reach USD 719.5 million by 2032, with an annual growth rate of 4.6% (FACT, 2022). Therapeutic proteins (e.g., antibodies, vaccines, enzymes, cytokines, and growth factors) account for nearly half of this market, followed by industrial proteins (e.g., technical enzymes) and research reagents (e.g., antibodies for protein detection and purification) (Markets & Markets, 2017). Generally, recombinant protein market analyses focus on the production of soluble active proteins. However, in recent years, the perception of IBs as an unwanted by-product has fundamentally changed, particularly in cases of high-value pharmaceutical products (Slouka et al., 2019). Given that IBs can be produced in high excess, IB-based processes are believed to significantly enhance production yields of recombinant proteins (Garcia-Fruitos et al., 2012; Berlec & Strukelj, 2013; Baeshen et al., 2015; Gupta & Shukla, 2017).

These biomaterials are designed such that they can release substantial amounts of functional recombinant protein with therapeutic effects directly from within cells (Vazquez et al., 2012). This biopharmaceutical delivery system led to the concept of "nanopills," and in this context, the range of possible IB applications in biomedicine has expanded (Vazquez et al., 2012). Delivery strategies are equally diverse, encompassing oral, transdermal, or targeted therapy (Talafová et al., 2013). Endotoxin-free IB-based biomaterials are therefore particularly promising, ensuring both functionality and biosafety while offering a cost-effective production advantage, as their estimated value is approximately 20 times lower than that of other protein-based drugs (Garcia-Fruitos et al., 2012; Rueda et al., 2014). However, market entry, like any veterinary drug, must overcome regulatory challenges in each country of application, including verification of compound effectiveness, safety, and consistency of potency and quality across batches.

### 3.7 Heterogeneity of available evidence

Data collected during this systematic review are heterogeneous, which prevents correlations between obtained variables. First, inherent differences between animal models (fish vs. mammals) and between species (e.g., bovine vs. porcine) generate divergent immune responses to similar IB formulations. For example, while TNFα IBs showed 75% protection against *Pseudomonas aeruginosa* in *D. rerio*, their effect in swine was marginal, suggesting limitations in interspecies translation. Additionally, variables such as age, physiological state, and sex of animals introduce greater variability, as observed in cattle, where females showed better responses to IBs against *Fasciola hepatica* than males.

Furthermore, the 16 included studies employ variable administration routes (oral, intraperitoneal, intramuscular) and non-comparable evaluation metrics (survival, gene expression, IgG levels). For example, while some studies report RPS, others only measure cytokines (Table 4). Seventy-one percent of studies present qualitative results (e.g., "significant increase in *il6*") without quantifiable data.

Methodological discrepancies are also evident, as the analyzed studies employ different expression systems (*E. coli* vs. *L. lactis*), variable purification methods, and non-comparable dosing schemes. This technical inconsistency hinders reproducibility, particularly because small changes in parameters such as pH or culture temperature alter IB structure and protein release capacity. The heterogeneity of current data limits quantitative analysis, but qualitative synthesis reveals useful patterns to guide future research.

## 4 Conclusions

The IB-based biomaterials reported in this study demonstrate remarkable versatility, both in types of proteins produced, ranging from host immune system proteins to highly antigenic proteins from pathogens, and in routes of administration. This adaptability has enabled their exploration for treating a wide range of diseases and pathogens relevant to aquaculture and livestock. Although the number of organisms evaluated so far is limited, studies encompass diverse sets of animal models, including rainbow trout, Senegalese sole, zebrafish for aquaculture, as well as cattle, sheep, pigs, and mice for livestock. In these models, these biomaterials have exhibited significant immunomodulatory effects and the ability to activate immune responses, which in some cases have led to effective disease control. Furthermore, *in vitro* and *in vivo* assays have confirmed that these biomaterials are non-toxic, efficiently endocytosed by cells, and capable of enhancing macrophage-mediated innate immune responses. This positions them as ideal candidates for designing immunomodulators or modular vaccines, potentially combined with adjuvants to further enhance their efficacy, a dual capacity that not only improves animal health but also offers long-term benefits by strengthening immune resilience.

However, despite these promising results, several challenges remain to be addressed. These include optimizing production processes to ensure consistency and scalability, reducing costs for large-scale applications, and further validating their efficacy across a broader range of species and pathogens. Additionally, more research is needed to fully understand the mechanisms underlying their immunomodulatory effects and to explore their potential in combination with other therapeutic strategies. Taken together, the versatility, safety, and immunomodulatory capacity of IB-based biomaterials make them a promising platform for improving animal health and productivity, one that, with continued research and development, could revolutionize disease management in the livestock and aquaculture industries, paving the way for more sustainable and efficient practices.

## Ethics approval and consent to participate

Not applicant.

## Consent for publication

All authors have approved the manuscript and agree with its submission.

## Funding

D.V.L. was supported by ANID Doctoral Scholarship No 21232344, and ANID VIU24P0043. D.T. was supported by ANID-Chile through a Fondecyt Iniciación No 11240684.

## CRediT authorship contribution statement

**Daniela Valentina López:** Writing – review & editing, Writing – original draft, Methodology, Funding acquisition, Formal analysis, Data curation. **José Gallardo-Matus:** Writing – review & editing, Conceptualization. **Débora Torrealba:** Writing – review & editing, Writing – original draft, Supervision, Funding acquisition, Data curation, Conceptualization.

## Declaration of competing interest

The authors declare that they have no known competing financial interests or personal relationships that could have appeared to influence the work reported in this paper.

## Data Availability

The data supporting the conclusions of this article will be made available by the authors, without undue reservation, to any qualified researcher.

## Appendix Supplementary materials

Image, application 1
