# Supplementary material for: Potential of inclusion bodies for disease control in aquaculture and livestock
Source: Vet Anim Sci. 2026 Aug 15;33:100820. doi: 10.1016/j.vas.2026.100820 (PMC13524869; doi:10.1016/j.vas.2026.100820)
Supplement: Supplementary file 1 [file mmc1.docx]

**Supplementary information**

**Supplementary Table 1.** Searching strategy and results by database.

| **Database** | **Applied filters** | **Date** |
| --- | --- | --- |
| PubMed | Include by species: other animals | 09/09/2025 |
| Web of Science | Exclude by research areas: biochemistry molecular biology (118), biochemical research methods (35), neuroscience (30), biophysics (13), chemistry multidisciplinary (12), medicine research experimental (11), endocrinology metabolism (8), engineering chemical (8), genetics hereditary (8), chemistry applied (6), chemistry physical (6), clinical neurology (6), medicine general internal (5), chemistry analytical (3), ophthalmology (3), rheumatology (3), urology nephrology (3), geriatrics (2), hematology (2), medical laboratory technology (2), oncology (2), pediatrics (2), peripheral vascular disease (2), physical applied (2), physics condensed matter (2), physics multidisciplinary (2), psychiatry (2), agricultural engineering (1), agriculture multidisciplinary (1), agronomy (1), anatomy morphology (1),chemistry organic (1), dentistry oral (1), dermatology (1), developmental biology (1), engineering environmental (1), entomology (1), environmental science (1), gastroenterology hepatology (1), orthopedics (1), plant science (1), respiratory system (1), substance abuse (1), surgery (1), polymer science (7), energy fuels (2), integrative complementary medicine (1).  Exclude by Sub-areas:  neuroscience neurology (32), chemistry (25), biophysics (13), research experimental medicine (11), endocrinology metabolism (8), engineering (8), tropical medicine (2) | 08/09/2025 |
| ScienceDirect | Exclude by publication title: neuromuscular disorders (57), journal of neurological science (25), seminars in arthritis and rheumatoides (31), journal of biological chemistry (167), protein expression and purification (149), biochemical and biophysical research (26), process biochemistry (29), journal of bioscience and bioengineering (23), archives in biochemistry and biophysics (17), biochemical engineering journal (15), methods in enzymology (14),  Exclude by subject areas:  agricultural and biological science (70), medicine and dentistry (30), chemical engineering (91) | 06/09/2025 |
| Scopus | Exclude by subject area: medicine (93), neuroscience (46), agricultural and biological sciences (46), chemistry (30), environmental science (24), materials science (22), engineering (20), physics and astronomy (9), energy (5), computer science (4), economics, econometrics and finance (3), nursing (2), earth and planetary sciences (2) psychology (1), mathematics (1), health professions (1), dentistry (1).  exclude by keyword: genetics (47), gene expression (24), dog (7), nerve cell (6), molecular genetics (6), huntington chorea (6), neurons (5), drosophila (5), case report (5), brain (5), bacterial gene (4). | 06/09/2025 |
